# Longitudinal multiparameter single-cell analysis of macaques immunized with pneumococcal protein-conjugated or unconjugated polysaccharide vaccines reveals distinct antigen specific memory B cell repertoires

**DOI:** 10.1371/journal.pone.0183738

**Published:** 2017-09-14

**Authors:** Bin Jia, Lisa K. McNeil, Christopher D. Dupont, Konstantinos Tsioris, Rachel M. Barry, Ingrid L. Scully, Adebola O. Ogunniyi, Christopher Gonzalez, Michael W. Pride, Todd M. Gierahn, Paul A. Liberator, Kathrin U. Jansen, J. Christopher Love

**Affiliations:** 1 Koch Institute for Integrative Cancer Research, Department of Chemical Engineering, Massachusetts Institute of Technology, Cambridge, Massachusetts, United States of America; 2 Pfizer Vaccine Research and Early Development, Pearl River, New York, United States of America; 3 The Ragon Institute of MGH, MIT and Harvard, Cambridge, Massachusetts, United States of America; Public Health England, UNITED KINGDOM

## Abstract

**Background:**

The efficacy of protein-conjugated pneumococcal polysaccharide vaccines has been well characterized for children. The level of protection conferred by unconjugated polysaccharide vaccines remains less clear, particularly for elderly individuals who have had prior antigenic experience through immunization with unconjugated polysaccharide vaccines or natural exposure to *Streptococcus pneumoniae*.

**Methods:**

We compared the magnitude, diversity and genetic biases of antigen-specific memory B cells in two groups of adult cynomolgus macaques that were immunized with a 7-valent conjugated vaccine and boosted after five years with either a 13-valent pneumococcal polysaccharide conjugate vaccine (13vPnC) or a 23-valent unconjugated pneumococcal polysaccharide vaccine (23vPS) using microengraving (a single-cell analysis method) and single-cell RT-PCR.

**Results:**

Seven days after boosting, the mean frequency of antigen-specific memory B cells was significantly increased in macaques vaccinated with 13vPnC compared to those receiving 23vPS. The 13vPnC-vaccinated macaques also exhibited a more even distribution of antibody specificities to four polysaccharides in the vaccine (PS4, 6B, 14, 23F) that were examined. However, single-cell analysis of the antibody variable region sequences from antigen-specific B cells elicited by unconjugated and conjugated vaccines indicated that both the germline gene segments forming the heavy chains and the average lengths of the Complementary Determining Region 3 (CDR3) were similar.

**Conclusions:**

Our results confirm that distinctive differences can manifest between antigen-specific memory B cell repertoires in nonhuman primates immunized with conjugated and unconjugated pneumococcal polysaccharide vaccines. The study also supports the notion that the conjugated vaccines have a favorable profile in terms of both the frequency and breadth of the anamnestic response among antigen-specific memory B cells.

## Introduction

Pneumococcal pneumonia remains a prevalent infectious disease globally, and particularly impacts children less than 2 years old and elderly adults more than 65 years old [1,2]. The pneumococcal capsular polysaccharides (PSs) confer virulence, and there are more than 90 known capsular serotypes of *Streptococcus pneumoniae* identified, of which at least 20 are responsible for most of the spectrum of pneumococcal disease [3, 4]. In the United States, two types of vaccines are currently available [5]. The first of these developed was a multivalent vaccine comprising 23 unconjugated PSs (23vPS; Pneumovax^™^); this vaccine is currently recommended for adults over 65 [6]. Vaccination with 23vPS provides some protection against invasive pneumococcal disease (IPD) in these populations, but its efficacy in the prevention of pneumococcal pneumonia has been unclear [7]. The lack of efficacy of unconjugated PS vaccines for infants led to the development of protein-conjugate pneumococcal vaccines [8, 9, 10], the most current of which, 13vPnC (also known as Prevnar13^®^), consists of 13 PSs conjugated to a mutant diphtheria toxoid (CRM_197_). This vaccine has been highly effective in preventing IPD for those pneumococcal serotypes included in the vaccine, and is currently recommended for children under the age of two and adults ≥ 65 years of age [2,6]. Protein-conjugated vaccines have generally been associated with superior immunological memory, greater levels of affinity maturation, greater levels of isotype-switching, and increased herd immunity relative to unconjugated PS vaccines [9]. The success of the 13vPnC vaccine in infants therefore has led to the hypothesis that vaccination with 13vPnC may provide better protection to adults than vaccination with 23vPS [10].

Several lines of evidence support the potential superiority of 13vPnC-vaccination for adults. A major limitation of the 23vPS vaccine is that its protection has been observed to wane following vaccination [11], possibly due to the decline in PS-specific antibody titers that occurs in 23vPS-vaccinated patients [12–16]. Additionally, several studies have observed that vaccination with 23vPS dampens subsequent immune responses to *S*. *pneumoniae* [13, 17–21], and 23vPS-vaccination has been reported to deplete PS-specific B cells [17]. Several studies directly comparing the immune responses induced by 23vPS and protein-pneumococcal polysaccharide conjugate vaccines have reported that protein-pneumococcal polysaccharide conjugate vaccines elicit superior B cell responses, opsonophagocytic activity, and/or antibody titers [17, 19, 22–24]. While another report observed no major differences between these vaccines [25], these studies collectively suggest that vaccination of older adults and other at risk populations with 13vPnC may be preferential to vaccination with 23vPS, and highlight the need for further studies to elucidate how these two vaccines differentially invoke protective immune responses.

The ambiguity generated from conflicting reports, the potentially pleiotropic effects of vaccination, and the uncertainty regarding the immunological factors that underlie differences in vaccine efficacy all underscore the need to measure multiple immunological parameters to identify improved correlates of protection. In addition to the size of the PS-specific memory B cell (MBC) pool and the titers and opsonizing activity of circulating antibodies, other aspects of the PS-specific MBC responses may correlate with protection following vaccination. These include the isotypes of antibodies produced, the breadth of antigens recognized, the clonal diversity of the PS-specific B cell response, and the presence of uniquely protective B cell clones. As traditional techniques such as ELISPOT that quantify PS-specific B cell responses do not allow for the isolation of PS-specific B cells, measuring many of these parameters has been historically challenging. Recent technological advancements, however, have increased the resolution and number of parameters that can be measured for a given cell population.

Microengraving is a technique for single-cell analysis in which cells are deposited in an array of sub-nanoliter wells (nanowells), and analyte secretion from individual cells is measured using fluorescently-labeled detection antibodies specific for analytes of interest [26]. In parallel, these cells can be stained with fluorescently-labeled antibodies specific for surface molecules of interest and imaged using fluorescence microscopy. Microengraving therefore enables the collection of phenotypic data from individual cells coupled with functional data regarding the cell’s secretory profile. An additional advantage of this method is that cells can later be isolated for further analysis [27], such as B cell receptor (BCR) sequencing [28], to examine the diversity of the B cell response and to identify specific B cells with uniquely protective properties. We have previously validated microengraving as an approach to identify antigen specific B cells [29–31].

Here, we have used microengraving to analyze the longitudinal recall responses elicited in adult nonhuman primates initially vaccinated with a heptavalent pneumococcal protein-conjugate vaccine. These studies indicate that distinctive differences exist in the responses elicited by booster vaccination with the 23vPS and 13vPnC vaccines. The frequencies and specificities of antibodies produced by both active antibody-secreting cells (ASCs) and ex-vivo activated memory B cells (MBCs) following a booster vaccination with either 23vPs or 13vPnC were characterized. These comparisons showed that vaccination with 13vPnC resulted in greater numbers of PS-specific B cells at 7 days post-vaccination, and a more even distribution of antigen specificities than the 23vPS vaccine. Sequencing the B cell receptors demonstrated that the PS-specific B cell responses induced by both vaccines had similar heavy-chain gene-family usage, and similar lengths of the complementary determining region 3 (CDR3). In addition, we demonstrated that microengraving can be utilized in the context of vaccination studies to increase the number of parameters measured relative to conventional ELISPOT assays, thereby obtaining a higher resolution of analysis. Future studies using this improved method of analysis may elucidate mechanisms that underlie the different immunological responses following 23vPS and 13vPnC vaccination, and facilitate the identification of novel and improved correlates of protection.

## Materials and methods

### Animals and immunization

Research was conducted in compliance with the Animal Welfare Act and other federal statutes and regulations relating to animals and experiments involving animals and adhered to principles stated in the Guide for the Care and Use of Laboratory Animals, National Research Council, 1996. The facility where the research was conducted is fully accredited by the Association for Assessment and Accreditation of Laboratory Animal Care International. Vaccination portions of the studies were conducted at Pfizer (Pearl River, NY). All studies were approved by the Pfizer Institutional Animal Care and Use Committee (S1 & S2 Files). All animal housing areas at Pfizer are continuously monitored for temperature and humidity using a state of art monitoring system, results are assessed regularly and recorded to ensure animal health and welfare. Animals were preferentially pair housed in stainless cages and provided environmental enrichment such as puzzle boxes, toys and television. Animals were supplied with primate diet throughout the study. In addition, animals were also provided with treats such as fresh fruits and vegetables. Water was available ad libitum throughout the study. The cynomolgus macaques used in this study were found to be filovirus-, STLV-1, SIV-, and Herpes B- antibody-negative in testing prior to initiation of the study. For blood sampling and immunizations, nonhuman primates were conscious and restrained in a veterinary-approved restraint device. Behavioral training is conducted with individual nonhuman primates to acclimate them to routine procedures such as phlebotomy and immunization. Throughout the study, animals were observed at least twice daily by husbandry, qualified scientific and/or veterinary staff. No adverse events due to immunization or blood sampling were noted. All procedures for animal care and housing are in accordance with the NRC Guide for the Care and Use of Laboratory Animals (1996) and the Animal Welfare Act as amended and standards incorporated in 9 CFR Part 3, 1991. No nonhuman primates were euthanized as a result of these studies.

All animal protocols employed in this study met the established Institutional Animal Care and Use Committee guidelines, and all animal work was conducted in an AALAC-accredited facility. Seven cynomolgus macaques (*Macaca fascicularis*) 7–8.5 years of age that had previously been immunized with 7-valent conjugated pneumococcal PS vaccines (7vPnC) five years prior were divided into two groups (S1 Table). One group (n = 3) was boosted by vaccination with 23vPS and another group (n = 4) was boosted by vaccination with 13vPnC. At 0, 7 and 28 days after boosting, PBMCs were isolated from whole blood and frozen in liquid nitrogen. Two animals in the 13vPnC group did not provide sufficient blood at day 28 to extract PBMCs and perform subsequent analysis. All animals were tetanus-toxoid antigen free and were housed in specific-pathogen free conditions.

### Cells, antigens and antibodies

Mouse hybridomas secreting antibodies against pneumococcal PS (serotypes 4, 6B, 14 and 23F), as well as tetanus toxoid-conjugated pneumococcal PS (TT-PS) (serotypes 4, 6B, 14 and 23F) were supplied by Pfizer Vaccine Research (Pearl River, NY). Each TT-PS or a mixture of the four serotypes of TT-PSs was labeled with Alexa Fluor carboxylic acid (Life Technologies Inc., Grand Island, NY). Polyclonal donkey anti-human IgG (H+L), polyclonal goat anti-human IgA and polyclonal goat anti-rabbit IgG were purchased from Jackson Immuno Research (West Grove, PA). Calcein Violet 405 live cell dye and Alexa Fluor 700 goat anti-rabbit IgG were purchased from Life Technologies (Grand Island, NY). Polyclonal goat anti-rhesus IgG (H+L) was obtained from Southern Biomedical Inc. (Birmingham, AL). PE-CD38 (clone OKT10) was provided by the NIH Nonhuman Primate Reagent Program. Alexa Fluor 647 CD20 (clone 2H7) was purchased from Biolegend (San Diego, CA). Alexa Fluor 488 mouse anti-human CD3 (clone SP34-2), purified mouse anti-human IgM (clone G20-127) and IgG (clone G18-145) were purchased from BD Biosciences (San Jose, CA). Alexa Fluor-labeled anti-human IgA, IgM and IgG were made by labeling corresponding antibodies with Alexa Fluor carboxylic acid (Life Technologies Inc., Grand Island, NY). Antigen-coated beads for luminex analysis were obtained from Pfizer. Polyclonal anti-human IgG for analysis of cloned antibodies was obtained from BD.

### Nanowell cytometry

Cryo-preserved cynomolgus PBMCs were thawed in a 37°C water bath, washed twice with R10 media [RPMI1640 (Corning Cellegro) containing 10% FBS (Atlanta Biologicals)], and incubated in 37°C for 1–2 hours (when used to detect ASCs) or incubated 3 days at 37°C in R10 supplemented with 2.5 μg/mL R848 (InvivoGen) and 1000 U/mL human recombinant IL-2 (Peprotech) to stimulate immunoglobulin secretion from memory B cells [32]. Approximately 5 x 10^5^ cells were washed and re-suspended in 200 μL R10. Subsequently, the cells were stained with Alexa Fluor 488 goat anti-mouse CD3, PE-CD38, and APC-CD20 for 20 min at room temperature. Calcein Violet was then added (1 μM final concentration) and incubations were extended for another 5 minutes. After washing twice with R10, cells were loaded into arrays of nanowells. Each array was 1 mm thick, was adhered to a 3”x1” glass slide, and consisted of 72x24 blocks of 7x7 nanowells (50 μm x 50 μm x 50μm with a 100 μm center-to-center distance). Before loading cells, arrays were treated with oxygen plasma for 2 min to sterilize and reduce hydrophobicity. After the cells were deposited in nanowells, the arrays were covered with a coverslip and imaged using an automated inverted epifluorescence microscope (Zeiss). Images were analyzed by AxioVision Rel. 4.7 software and custom imaging analysis software (Enumerator and EnumCytoViz).

### Microengraving

Microengraving experiments were performed as previously described [26,33]. After imaging by fluorescence microscopy, arrays were washed with R10 and covered with a poly-L-Lysine coated glass slide pre-coated with donkey anti-human IgG, goat anti-rhesus IgG and donkey anti-rabbit IgG. Rabbit serum was added to each sample (diluted 1:10,000) prior to microengraving to serve as a positive control signal. The assembled array and slide complex were sealed tightly in a hybridization chamber (Agilent, Englewood, CO). The chamber was incubated at 37°C with 5% CO_2_ for 2 h (printing). After printing, the assembled array and slide were immersed in R10 media and the slide was gently separated from the array. The slide was then blocked with non-fat milk (3% w/v in PBS) containing 10 μg/mL cell wall PS and pneumococcal PS serotype 22F for 30 min at 25°C to absorb antibodies against these two antigens and then blotted with Alexa Fluor-labeled IgG, IgM, IgA and TT-PS (2.5 μg/mL) for 45 mins. After extensive washing, the glass slides were scanned using a Genepix 4200AL microarray scanner (Molecular Devices). Genepix 6.1 and Crossword [34] software were used to extract the mean fluorescence intensity (MFI) of each spot.

### Single-cell RT-PCR

Single cells in nanowells identified by microengraving were retrieved using an automated micromanipulation system (CellCelector, AVISO GmbH) and deposited into 96-well PCR plates containing 10 μL of 1x first strand buffer from SuperScript III RT-PCR kit (Life Technologies Inc.) and 20U of RNase Inhibitor (Promega). Single-cell RT-PCR was performed following the protocol of Wang et al. [35]. The primers for the first PCR were designed based on rhesus immunoglobulin sequences obtained from the rhesus immunoglobulin database (http://www.kcl.ac.uk/immunobiology/Mac_ig/). The primers for the second PCR were borrowed from Kuwata T. et al. [36]. After picking, cells were stored at -80°C before first strand DNA synthesis. For the reverse transcription (RT) reaction, 3 μL of 10% NP-40 (G-Biosciences), 0.5 μg random hexamer primers (Promega) and 1 μL of 10 mM dNTPs were added to the PCR wells. The plates were then incubated at 65°C for 10 min for cell lysis and then at 25°C for 3 min. After lysis, 2.25 μL of 5x first strand buffer, 1 μL of superscript III (200U/μL) and 1 μL of 0.1M DDT were added into each well and the RT reaction was performed at 37°C for 60 min followed by a 70°C incubation for 10 min to deactivate the reverse transcriptase. The two-round PCR for amplifying variable regions of immunoglobulin heavy and light chains was performed using the Roche FastStart High Fidelity PCR system. The first PCR (50 μL) reaction mixture for the first round of PCR for heavy and light chains contained 5 μL of 10x buffer (18 uM Mg^2+^), 1 μL of 10 mM dNTP mix, 0.5 μL of forward or reverse primer mix (10μM each primer), 0.1 μL of (5U/μL) enzyme, and 5 μL of template from the RT reaction. Cycling parameters of the first PCR were 94°C/45s, 45°C/45s, and 72°C/105s for 3 cycles, followed by 94°C/45s, 50°C/45s, and 72°C/105s for 30 cycles. The second PCR contained 5 μL of 10xPCR buffer (18 mM Mg2+), 1 μL of 10 mM dNTP, 0.5 μL of forward and reverse primer mix (10 μM each), and 3 μL of template from the first PCR. Cycling parameters for the second PCR were 94°C/45s, 50°C/45s and 72°C/105s for 35 cycles. After the second PCR, 5 μL of product was run on a 2% agarose gel to separate PCR products on the basis of their size. PCR products with expected sizes for variable regions (~500 bp) were SANGER sequenced. Sequences were compared with entries at the rhesus germinal sequence database of IMGT to extract variable region sequences.

### Statistical analysis

The average numbers of PS-specific MBCs in conjugated and unconjugated vaccine groups were compared by Fisher’s test. The numbers entered into the contingency table were determined by subtracting the numbers of positive events from the numbers of total events to determine the numbers of negative events. These numbers (the total number of antigen-specific B cells and the total number of non-antigen specific B cells) were then compared for each category using a contingency table. The distributions of PS-specific MBCs against each antigen were compared by 2XN Fisher’s test. A contingency table was used in which each column represents the vaccine group (13vPnC or 23vPS) and each row represented the number of events that were identified specific for each antigen (PS4, 6B, 14, 23F). The heavy-chain gene-family usages of PS-specific MBCs were compared by Fisher’s Exact Test.

## Results

### Study design and analytical approach

A group of seven cynomolgous adult macaques who had been previously vaccinated five years prior with a heptavalent PS conjugate vaccine (7vPnC) were divided into two groups to receive either the unconjugated 23vPS (N = 3) or the conjugated 13vPnC (N = 4) vaccine (S1a Fig). Because understanding the effects of pneumococcal vaccination on elderly individuals is an area of particular interest, all macaques were adult (7–8.5 years of age) at the time of booster vaccination. Due to the high likelihood of previous exposure to pneumococcal antigens in elderly patients (through vaccination, infection, or carriage), analyzing the recall response to booster vaccination in the animal model provide a model of this response. Blood was drawn pre-vaccination (day 0), and twice post-vaccination on days 7 and 28 (S1 Table). Cryopreserved peripheral blood mononuclear cells (PBMCs) were used to characterize both ASCs and MBCs (S1b Fig). MBCs were defined based on their ability to secrete antibodies in response to ex vivo stimulation [32]. To examine the phenotypes of total B cells ex vivo, we used biocompatible microchips containing arrays of nanowells to perform single-cell on-chip cytometry by epifluorescent microscopy. In addition, we employed the nanowell devices for microengraving to assess the number of PS-specific B cells, their isotypes, and the genes encoding the immunoglobulin (Ig) variable regions of the heavy (V_H_) and light (V_L_) chain.

Microengraving is a method previously developed in our laboratory for printing protein microarrays wherein each element of the array comprises captured secreted proteins from single-cells in each nanowell [26]. Using this technique, multiplexed analyses of Ig isotypes, specificities, and relative affinities of the antibodies produced by single B cells can be determined [26,37,38].

We adapted an analytical process using two arrays of nanowells to characterize the PBMC samples. A portion of the PBMCs were labeled for viability and surface-markers (CD3, CD20, CD27, CD38), deposited into the nanowells, imaged by automated epifluorescence microscopy, and subsequently used for microengraving to determine the total numbers and isotypes of Ig-secreting cells (IgM, IgG, IgA), as well as the percentage of PS-specific B cells.

A second portion of the cells were deposited into a second array of nanowells, and used for microengraving to assess the breadth of specificities of four capsular PSs common to both vaccines as well as the original priming vaccine (serotypes 4, 6B, 14, 23F). For PS-specific antibodies of interest, the corresponding B cell was retrieved by automated micromanipulation [37,39] for subsequent single-cell RT-PCR to recover the V_H_ and V_L_ gene segments. We applied this single-cell analysis method directly to previously-frozen PBMCs, and to activated MBCs for each time point for each macaque.

Polysaccharides are inherently difficult to directly label with fluorescent dyes. Therefore we opted to label tetanus toxoid-conjugated PSs (TT-PS). Specificities of ASCs were determined using Alexa Fluor-labeled TT-PSs of serotypes 4, 6B, 14 and 23F in four separated fluorescent channels. This approach was possible since all immunized macaques were TT naive. Furthermore, when macaque PBMCs were examined, no cells were found to be specific for multiple TT-PS conjugates, confirming that the antigen-specific cells detected were in fact specific for the PSs rather than the tetanus toxoid. This technique was also validated using murine hybridomas that secrete antibodies specific for each PS serotype (S2 Fig). Lastly, specificity was demonstrated by retrieving individual cells and cloning antibodies, 75% of these antibodies were found to be specific for pneumococcal antigens by Luminex analysis (S2 Table).

**Fig 1:**
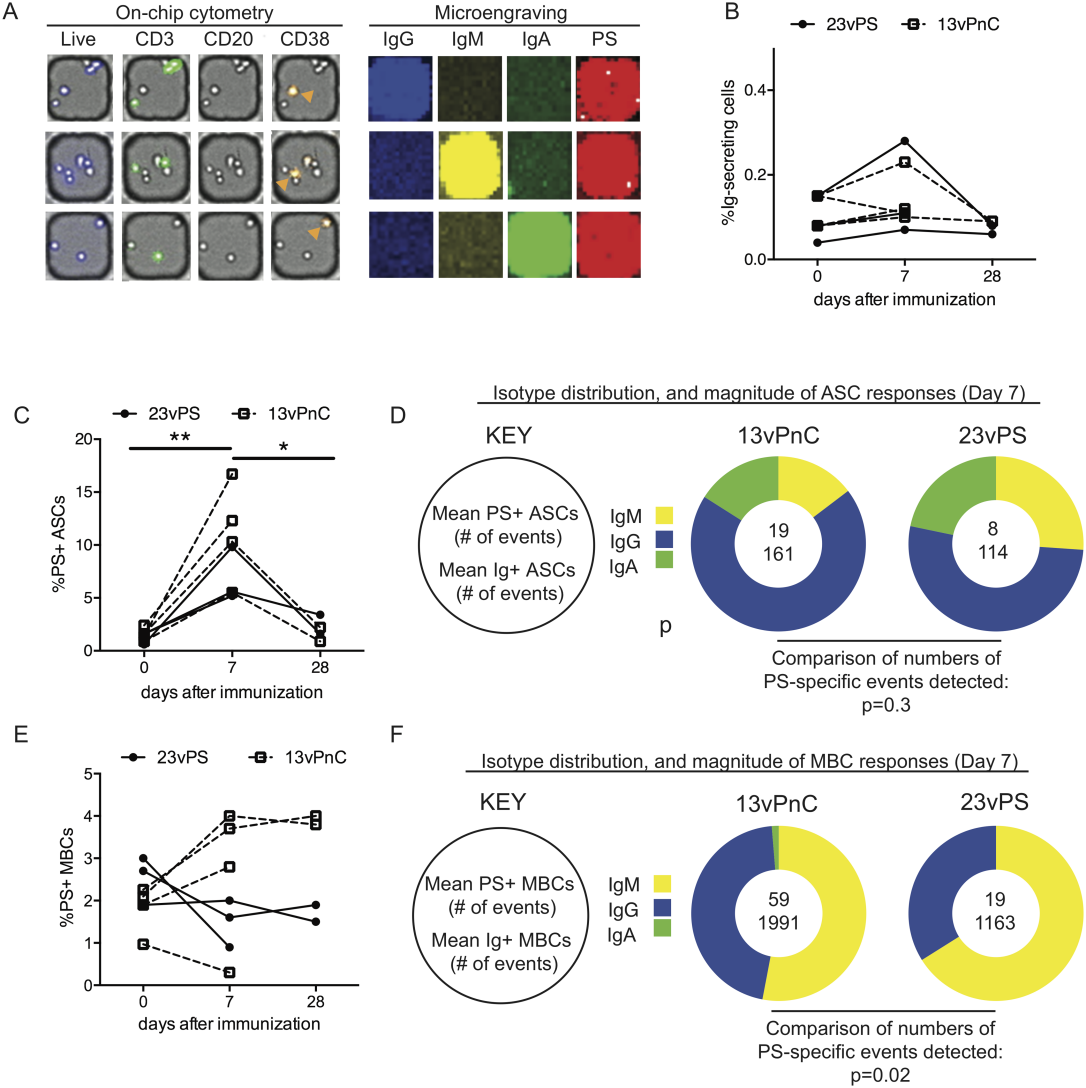
The average frequency of polysaccharide-specific antibody-secreting cells and memory B cells. (A) Representative images of 3 nanowells to illustrate the combination of on-chip imaging cytometry (left) with microengraving (right) in the analysis of circulating ASCs. Each horizontal row shows one nanowell (left) and the corresponding print obtained by micro-engraving (right). For images obtained by on-chip cytometry, brightfield images of each nanowell are overlaid with different fluorescence images demonstrating cell viability (violet), expression of CD3 (green), expression of CD20 (not detected) and expression of CD38 (yellow) for the cells in each nanowell. ASCs (Live, CD3^-^CD20^-^CD38^+^) are identified by orange arrows. Images obtained following microengraving to measure the antibody secretion from each ASC are shown on the right. Secretion of IgG (blue), IgM (yellow), and IgA (green) were measured. Antigen specificity was determined using a fluorescently-labeled PS-protein conjugate (red). Images obtained for on-chip cytometry were obtained using AxioVision Rel. 4.7 (Zeiss) and spots from microengraving were analyzed using Genepix 7.0 (Molecular Device). (B) Kinetics of the PS-specific circulating ASC response, represented as percent of PS-specific antibody secreting cells among total PBMCs from individual macaques boosted with either 13vPnC or 23vPS vaccines at days 0, 7 and 28 post-vaccination. Frequencies of PS-specific ASCs were determined by dividing the total number of PS-specific spots (specific for PS4, 6B, 14, and 23F) by the total number of PBMCs on the nanowell array. The total number of PBMCs was obtained by counting the total live cells using in-house software “Enumerator.” (C and E) Kinetics of the ASC (C) and MBC responses (E) represented as the percentage of Ig-secreting cells that are specific for PS4, 6B, 14, and 23F, as measured by microengraving coupled with on-chip cytometry. Frequencies of PS-specific ASCs and MBCs from day 0, 7 and 28 in each macaque are shown. The average frequency of PS-specific ASCs among both groups at day 0 was compared with the average frequency of PS-specific ASCs at day 7. The average frequency of PS-specific ASCs among both groups at day 7 was also compared with the average frequency of PS-specific ASCs at day 28 (C). (D and F) Average numbers of ASCs (D) and MBCs (F) detected in the 13vPnC-vaccinated and the 23vPS-vaccinated groups at day 7 post-vaccination, and the isotype distribution of the PS-specific ASC (D) and MBC (F) responses (displayed in pie charts). The top number within each pie chart indicates the number of PS-specific ASCs (D) or MBCs (F) detected and the bottom number is the total number of ASCs (D) and MBCs (F). The total numbers of ASCs and MBCs were calculated by adding the positive spots of IgG, IgM and IgA identified by microengraving. Yellow represents the fraction of IgM, blue represents IgG, and green represents IgA. ASC and MBC responses induced by the 23vPS or 13vPC vaccines were compared using Fisher’s test.

We analyzed PBMCs by combining fluorescent microscopy (used to identify cellular surface molecules) with microengraving (used to identify antibody secretion events of PS-specific B cells). To compensate for the low frequency of ASCs in PBMCs (<1%) and to optimize the detection of these events, we loaded the nanowell devices (each of which contains 1x10^5^ wells) with 0.5x10^6^ cells, resulting in multiple cells per well, of which no more than one was an ASC on average (defined by their CD3^-^CD20^-^CD38^+^ phenotype [40,41]). As expected, all PS-specific spots were found to also stain positive for one of the three isotypes examined (IgG, IgM, or IgA) (Fig 1A).

### The average frequency of polysaccharide-specific memory B cells was significantly higher in the 13vPnC vaccine group compared to the 23vPS vaccine group

To compare the PS-specific B cell responses upon boosting with unconjugated or conjugated vaccines, we first determined the frequencies of PS-specific ASCs in total PBMCs in un-boosted macaques, which was between 0.1–0.2% (Fig 1B). The frequency of PS-specific ASCs increased 5 to 10 fold in both groups of vaccinated macaques at day 7 after boosting compared with the level before immunization (Fig 1C). The frequencies of PS-specific ASCs in some macaques in the 13vPnC vaccine group trended higher (Fig 1C), but no significant difference (Fisher’s test; p = 0.3) was observed between the mean frequencies of the two groups. This outcome may have been due to the low numbers of PS-specific ASCs identified (Fig 1D).

Following booster vaccination with the 13vPnC vaccine, the frequency of PS-specific MBCs in the 13vPnC vaccine group (reported here as % of total MBCs) increased approximately 1–2% from day 0 to day 7 in 3 of 4 macaques (Fig 1E). In contrast, the frequency of PS-specific MBCs in the 23vPS vaccine group decreased approximately 1–2% in two macaques and remained constant in the third macaque. The decrease in PS-specific MBCs is consistent with the notion that MBCs are depleted upon boosting with the 23vPS vaccine. In a majority of the macaques, the frequency of PS-specific MBCs at 28 days after boosting was similar to the frequency observed at day 7. Comparing the average frequencies of PS-specific MBCs 7 days after boosting suggested that immunization with the 13vPnC vaccine led to a greater expansion and class switching of PS-specific B cells than the 23vPS vaccine (Fisher’s test; 59 vs 19; p = 0.02) (Fig 1F). Enhanced IgM to IgG class switching could suggest that the 13vPnC vaccine induces a higher degree of affinity maturation than the 23vPS vaccine (Fig 1F).

### Immunization with the 13vPnC vaccine induces a significantly broader response against input polysaccharides than the 23vPS vaccine

**Fig 2:**
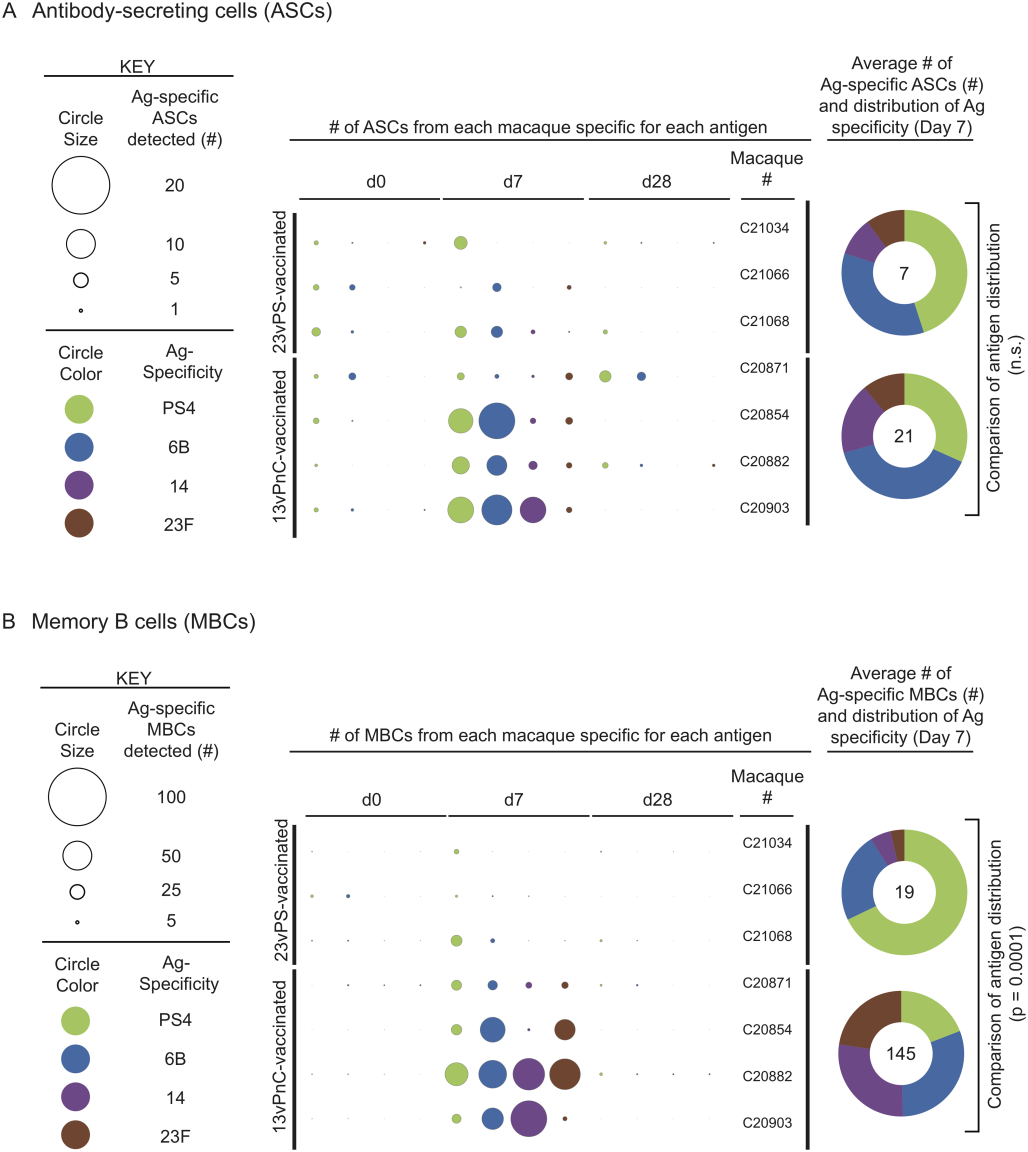
The distribution of polysaccharide-specific antibody-secreting and memory B cells against the four polysaccharides. The number of PS-specific ASCs (A) and MBCs (B) specific for each antigen at days 0, 7 and 28 post-vaccination are depicted. The radius of each circle in the center panel is proportional to the number of PS-specific ASCs or MBCs detected, and the antigen specificity is indicated by the color of each circle. The antigen distribution for all ASCs and MBCs within each vaccine-group are depicted in pie charts. Numbers in the center of the pie charts indicate the total numbers of ASCs or MBCs detected. The distribution of antigen specificity was compared using a 2XN Fisher’s test.

Pneumococcal PS vaccines include multiple PS antigens (13 in the 13vPnC and 23 in the 23vPS vaccine) to protect against different serotypes of *S*. *pneumoniae*. Induction of an evenly distributed immune response towards each PS antigen included in a vaccine is therefore desirable, to maximize the number of *S*. *pneumoniae* serotypes against which an individual will be protected. To dissect the antibody response for a subset of PSs shared between the two vaccines, we compared the relative distribution of PS-specific B cells for PS serotypes 4, 6B, 14 and 23F. In the unconjugated 23vPS vaccine group at day 7 post-immunization, macaques had more B cells specific to serotypes 4 and 6B in circulation than those recognizing serotypes 14 and 23F, as measured by the total numbers of ASCs (Fig 2A) and MBCs specific for each antigen (Fig 2B). In contrast, the data suggests that in the 13vPnC vaccine group, the macaques generated a more evenly distributed response against the four antigens measured. This even distribution of antigen specificities was particularly apparent in the PS-specific MBC compartment in macaques C20882 and C20871 (Fig 2B), and the difference in the distribution of responses against the four antigens between the unconjugated 23vPS and conjugated 13vPnC vaccine groups was significant on day 7 in the MBC compartment (2XN Fisher’s test; p = 0.0001, Fig 2B).

### Heavy chain gene usage and mean length of the CDR3 in memory B cells were not significantly different between the 13vPnC and the 23vPS vaccine groups

**Fig 3:**
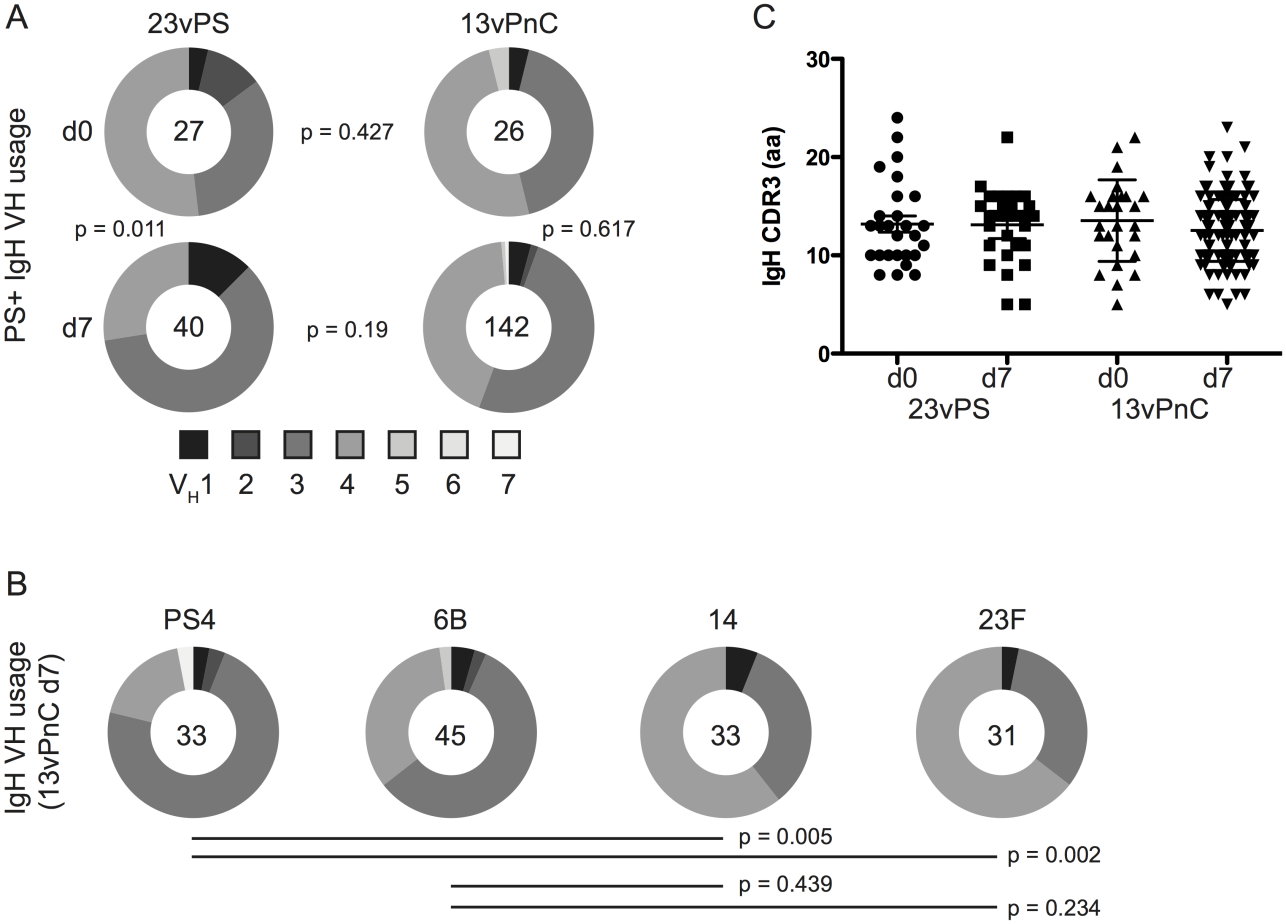
The heavy-chain gene-family usage and CDR3 length of polysaccharide-specific memory B cells. Sequences recovered from PS-specific MBCs were analyzed by IMGT/HighV-QUEST using the rhesus macaque immunoglobulin database. The heavy chain alleles of each B cell were generated by RT-PCR. (A) Heavy-chain gene-family usage at day 0 and 7 after immunization with 23vPS or 13vPnC. The number in the pie chart is the average number of variable sequences recovered from PS-specific MBCs. Pairwise statistical analysis was performed by Fisher’s Exact Test. (B) Distribution of heavy-chain gene-family usage for each PS serotype from PS-specific MBCs at 7 days following immunization with 13vPnC. The number in the pie chart is the average number of PS-specific MBCs against each antigen. Statistical analysis was performed by Fisher’s Exact Test. (C) The average CDR3 amino acid length of PS-specific MBCs at 0 and 7 days after immunization.

To compare the diversity of the PS-specific B cell repertoire between the two groups of immunized macaques, we retrieved single B cells from nanowells identified as PS-specific ASCs or activated MBCs. The immunoglobulin (Ig) variable region coding sequences from the identified B cells were recovered by single-cell RT-PCR (see supplementary material). We assigned germline rhesus macaque variable heavy chain (V_H_) gene-family sequences from the the IMGT database to the recovered Ig sequences [42]. We found no significant differences (Fisher’s Exact Test; p = 0.427 at day 0 and p = 0.19 at day 7 after immunization) in V_H_ gene-family usage between the 23vPS and 13vPnC- vaccine groups. However, a significant increase in usage of the V_H_1 genes in the 23vPS-vaccinated group was observed at day 7, relative to day 0 (Fisher’s Exact Test; p = 0.011, Fig 3A). In contrast, the data suggest that no vaccine-induced change in heavy-chain gene-family usage was apparent within the total PS-specific B cell population of the 13vPnC-vaccinated group (Fisher’s Exact Test; p = 0.617). When the V_H_ gene usage was compared across the individual antigens of the 13vPnC vaccine group, significant differences were found between the PS4-specific vs. PS14-specific B cells (Fisher’s Exact Test; p = 0.005) and the PS4-specific vs. PS23F-specific B cells (Fisher’s Exact Test; p = 0.002). These findings suggest that certain antigens may preferentially bind antibodies originating from specific V_H_ germline sequences (Fig 3B).

We compared the usage of V_H_ alleles with heavy chain joining (J_H_) alleles for the PS-specific MBCs (S3 Table). We observed at day 7 after immunization, 12 V_H_ chain alleles to be paired with 7 J_H_ chain alleles in the 13vPnC-vaccinated group, while only 7 different V_H_ chain alleles paired with 7 J_H_ chain alleles in 23vPS-vaccinated group (S3 Fig). Similar observations were also obtained when the V_H_ and J_H_ gene usage of PS-specific ASCs were examined (S3 Fig). These observations, however, were limited by the high variability in the numbers of antibody sequences recovered from the protein-conjugated and unconjugated vaccine groups.

The CDR3 loop is one of the three domains in the variable region of an antibody that directly binds antigen and experiences the highest degree of SHM. Changes in the length of the CDR3 region are indicative of the antibody repertoire diversity [43–46]. We compared the average lengths of heavy chain CDR3 regions from day 0 to day 7 between the two vaccine groups. Although we observed a higher level of class-switched B cells in the MBC compartment at day 7 in the 13vPnC group (Fig 1F) compared to the 23vPS group, we found no significant differences in CDR3 lengths between the two groups (Fig 3C) (Reported as mean ± 95% CI). Antibody VH and Vl sequences are provided in Supporting Information section (S4 Table).

## Discussion

We performed a longitudinal analysis of the PS-specific memory B cells (MBCs) and antibody-secreting cells (ASCs) following immunization of nonhuman primates with unconjugated and protein-conjugated pneumococcal PS vaccines by microengraving. We evaluated multiple immunological parameters simultaneously including the frequency, isotypes, and B cell receptor sequences of PS-specific B cells. Unlike B cell ELISPOT assays which normally involve culturing 10^5^ cells in a volume of 100 μL, the nanowell-based assay permits the culture and analysis of 1 cell in a volume of ~100 pL. This reduced volume results in a 10^6^ fold higher concentration of analytes from any given cell, thereby increasing the sensitivity of the assay [47]. Additionally, this increased concentration makes it possible to shorten the incubation time during which analytes are captured to 1–2 hours, compared with the overnight incubations required for most ELISPOT assays [48]. Furthermore, microengraving enables the recovery of analyzed cells for further genetic analysis. This work represents the first application of microengraving for the longitudinal analysis of PS-specific B cell repertoires and demonstrates the potential utility of this technique in human clinical vaccine trials.

While recent data from large-scale clinical trials with approximately 1,000 elderly adults indicate that pneumococcal PS-conjugate vaccines have immunological advantages over unconjugated PS vaccines based on the measurement of opsonophagocytic (OPA) titers [23], the underlying mechanisms of this phenomenon are still unclear. Marginal zone B cells (CD27^+^IgM^+^) [49] recognize PS antigens independently of T cell help, and are thus likely to respond well to the 23vPS vaccine. In contrast, conventional B cells, which depend on help from T cells, are more responsive to the protein-conjugated vaccines, since protein antigens elicit T cell responses far more effectively than PS antigens [9]. Indeed, previous studies have reported that the CD27^+^IgM^+^ population was activated by immunization with the 23vPS vaccine [50]. We also observed that vaccination with 13vPnC induced more class-switched (IgG^+^, IgA^+^) B cells than vaccination with 23vPS, which is consistent with this model. Furthermore, we observed that the protein-conjugated vaccine expanded the PS-specific MBCs whereas the unconjugated vaccine depleted PS-specific MBCs. This result is also consistent with current models in which conventional B cells form MBCs more effectively than marginal zone B cells.

Previous studies have examined the sequences of pneumococcal-specific immunoglobulins induced by vaccination from healthy human adults [51,52]. These studies observed pneumococcal-specific variable region sequences to predominantly utilize VH3 regions, while some utilized VH1. These findings stand in contrast to our results, in which in addition to VH3, the VH4 region was also widely used (Fig 3). This additional use of the VH4 region could be attributable to differences in the immune response of humans compared to non-human primates as used in the present study.

The diversity of B cell repertoires correlates with the level of protection induced by immunization [53]. For example, elderly individuals have less clonal diversity and less isotype diversity in their B cell responses to influenza vaccination than younger controls, and this phenomenon may contribute to the decreased efficacy of influenza vaccination among elderly patients [53,54]. In the analysis of the PS-specific MBCs against individual antigens, we found that the protein-conjugated vaccine induced a broader response to the included antigens than the unconjugated 23vPS vaccine. Additionally, macaques boosted with 13vPnC had greater numbers of PS-specific B cells than those boosted with 23vPS. Collectively, these findings support the notion that vaccination with 13vPnC may provide a more balanced immune response in terms of both the frequency and breadth of antigen specific B cells than 23vPS to elderly individuals.

## Conclusion

Our results confirm that differences in the frequency and distribution of polysaccharide-specificity can be recognized in the MBC repertoires from nonhuman primates immunized with either unconjugated or protein-conjugated pneumococcal polysaccharide vaccines. The results are consistent with the notion that conjugated vaccines such as 7vPnC and 13vPnC have a favorable response in terms of the frequency and breadth of the PS-specific MBC response compared to unconjugated vaccines such as 23vPS. In addition, we observed similar results in the ASC compartment in some macaques, though the observations were limited by the low number of cells recovered. Improving the power of these studies can be accomplished by increasing the number of cells analyzed to increase the number of rare ASCs detected. Identifying higher numbers of ASCs will allow for the elucidation of a more complete picture of the B-cell response to pneumococcal polysaccharide vaccines, as well as other vaccines. Nevertheless, the methodology developed here provides a strong foundation for future in-depth analysis of antigen-specific B-cell repertoires in both nonhuman primate immunogenicity studies, as well as in human vaccine trials.

## Supporting information

S1 Fig Flowchart of experiments. (A) Grouping and immunization of macaques. (B) Assays applied to the PBMCs isolated from immunized macaques. (C) Integrated process for analysis of PBMCs by microengraving and single-cell cytometry and the output of data.
(PDF)

S2 Fig Validation of Alexa Fluor-labeled PS4, 6B, 14 and 23F using PS-specific mouse hybridomas. Images (A and C) and mean fluorescent intensity (B and D) obtained from signals of PS-specific antibodies secreted from PS-specific hybridomas by Alexa Fluor 555-labeled PS4 and Alexa Fluor 647-labeled PS23F (A and B) and Alexa Fluor 488-labeled PS14 and Alexa Fluor 594-labeled PS6B (C and D) using microengraving as well as live cell imaging.
(PDF)

S3 Fig Immunization with 13vPnC compared to 23vPS vaccine incurred a broader V and J chain usage in forming antigen-binding sites. Sequences recovered from PS-specific ASCs or MBCs were analyzed by IMGT/HighV-QUEST using the rhesus macaque immunoglobulin database. The variable (V) and joining (J) chain alleles of each B cell were analyzed. The heatmaps show the V and J chain usage for PS-specific ASCs and MBCs against serotype 4, 6B, 14 and 23F at day 7 following immunization with 23vPS (A) or 13vPnC (B). The number of B cells using the same V and J chain combination is illustrated by the color intensity and number in each grid.
(PDF)

S1 Table Ages of macaques used for this study. (PDF)

S2 Table Luminex analysis confirmed antigen-specificity of isolated B cells. After isolation of individual antigen-specific B cells, heavy and light chain sequences were isolated and expression vectors were developed. 293T cells were co-transfected with heavy and light chains from a single B cell. Lysates from 293T cells were captured with luminex beads coated with the PS4, PS14, PS6B or PS23F antigens, and probed with PE-conjugated anti-human Ig antibodies. PE fluorescence was measured. Fluorescence intensities (measured in arbitrary units) for each cloned antibody and each antigen are shown.
(PDF)

S3 Table V_H_ and J_H_ allele usage and CDR3 sequences from antigen-specific antibodies. (XLSX)

S4 Table Table of antibody VH and VL sequences. (ZIP)

S1 File Original AUP approval. (PDF)

S2 File Updated AUP approval. (PDF)
