# Supplementary figures and images for: Longitudinal multiparameter single-cell analysis of macaques immunized with pneumococcal protein-conjugated or unconjugated polysaccharide vaccines reveals distinct antigen specific memory B cell repertoires
Source: PLoS One. 2017 Sep 14;12(9):e0183738. doi: 10.1371/journal.pone.0183738 (PMC5598952; doi:10.1371/journal.pone.0183738)

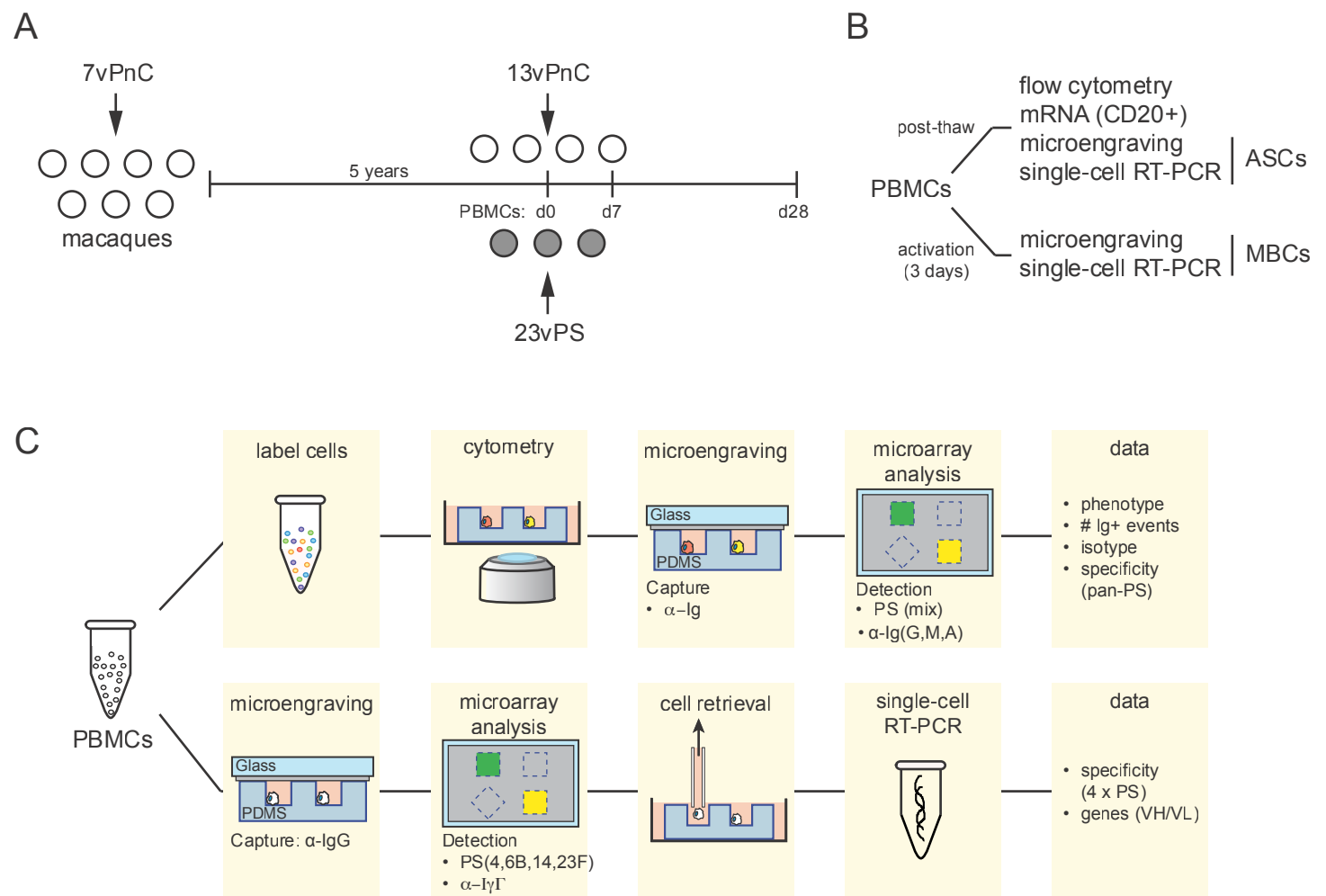

Supplemental Figure 1

Supplement: S1 Fig — (A) Grouping and immunization of macaques. (B) Assays applied to the PBMCs isolated from immunized macaques. (C) Integrated process for analysis of PBMCs by microengraving and single-cell cytometry and the output of data. (PDF) [file pone.0183738.s001.pdf]

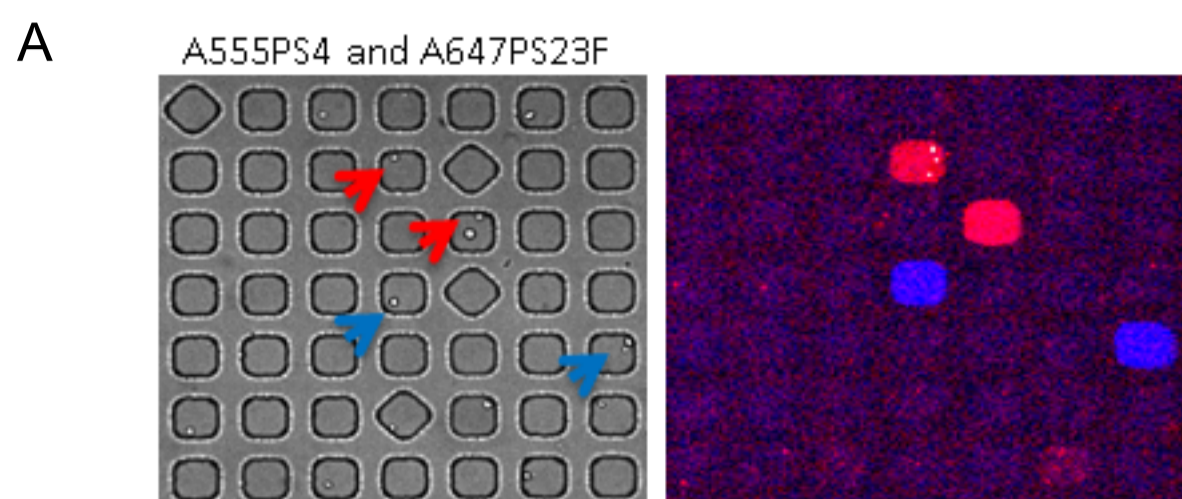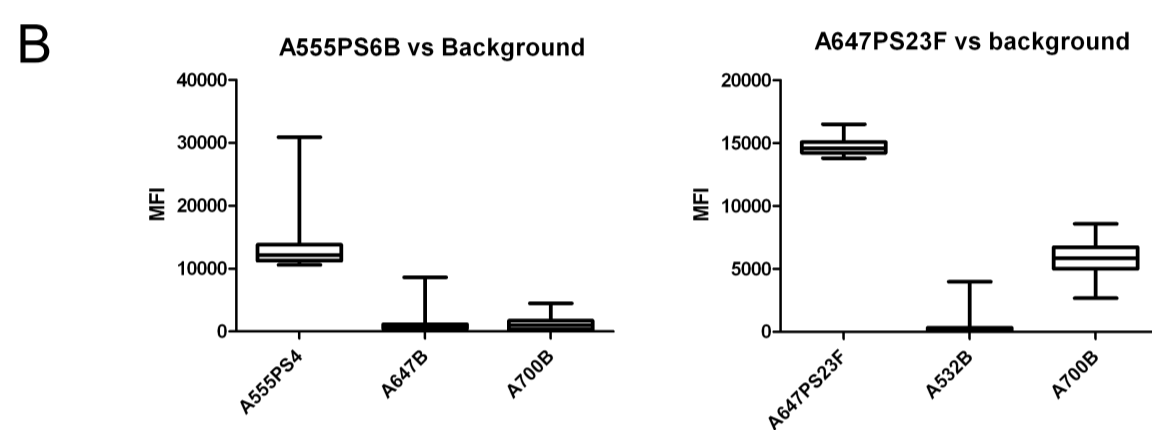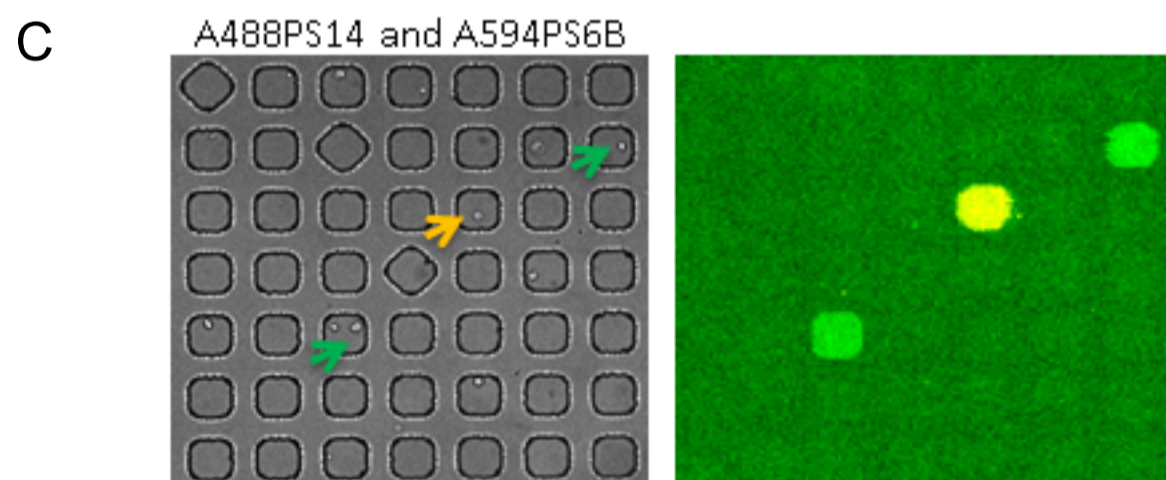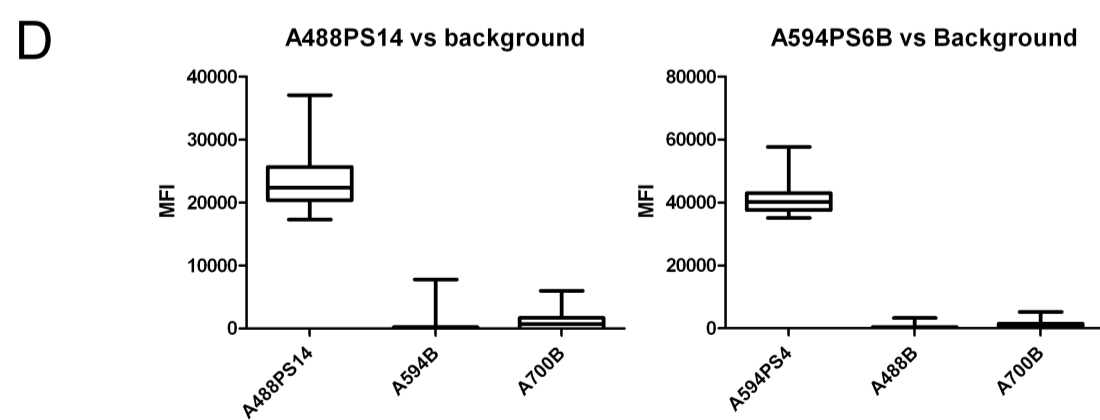

Supplemental Figure 2

Supplement: S2 Fig — Images (A and C) and mean fluorescent intensity (B and D) obtained from signals of PS-specific antibodies secreted from PS-specific hybridomas by Alexa Fluor 555-labeled PS4 and Alexa Fluor 647-labeled PS23F (A and B) and Alexa Fluor 488-labeled PS14 and Alexa Fluor 594-labeled PS6B (C and D) using microengraving as well as live cell imaging. (PDF) [file pone.0183738.s002.pdf]

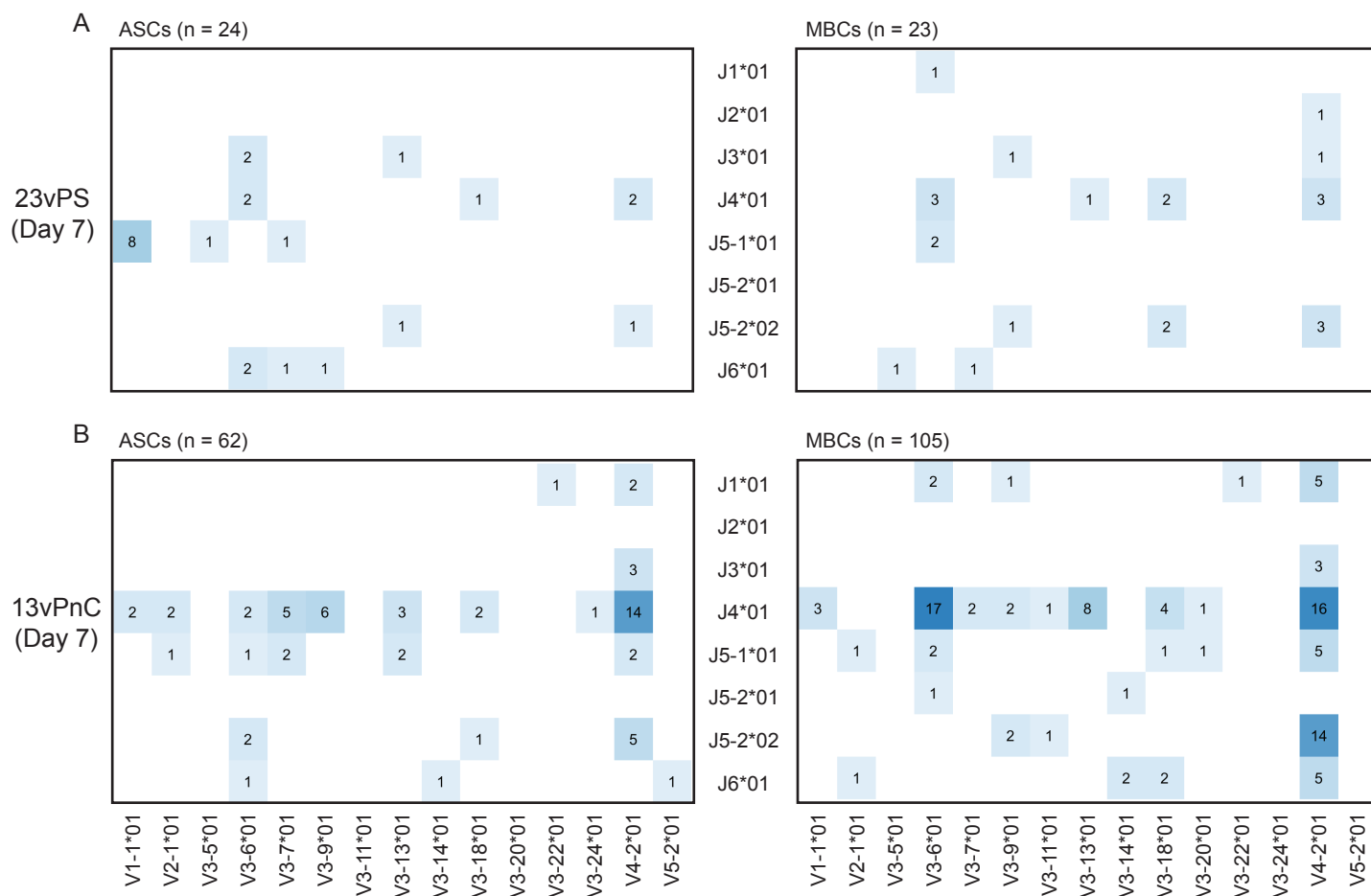

Supplemental Figure 3

Supplement: S3 Fig — Sequences recovered from PS-specific ASCs or MBCs were analyzed by IMGT/HighV-QUEST using the rhesus macaque immunoglobulin database. The variable (V) and joining (J) chain alleles of each B cell were analyzed. The heatmaps show the V and J chain usage for PS-specific ASCs and MBCs against serotype 4, 6B, 14 and 23F at day 7 following immunization with 23vPS (A) or 13vPnC (B). The number of B cells using the same V and J chain combination is illustrated by the color intensity and number in each grid. (PDF) [file pone.0183738.s003.pdf]
