# Supplementary material for: Longitudinal multiparameter single-cell analysis of macaques immunized with pneumococcal protein-conjugated or unconjugated polysaccharide vaccines reveals distinct antigen specific memory B cell repertoires
Source: PLoS One. 2017 Sep 14;12(9):e0183738. doi: 10.1371/journal.pone.0183738 (PMC5598952; doi:10.1371/journal.pone.0183738)
Supplement: S1 Table — (PDF) [file pone.0183738.s004.pdf]

Animal	Date of Birth	Age
C20871	6/17/04	7yrs, 2mo
C20854	4/20/03	8yrs, 4mo
C20882	3/19/04	7yrs, 5mo
C20903	1/2/2003	8.5yrs
C21034	1/25/04	7.5yrs
C21066	10/23/03	8yrs
C21068	11/27/03	8yrs

Supplemental Table 1
