# Supplementary material for: Longitudinal multiparameter single-cell analysis of macaques immunized with pneumococcal protein-conjugated or unconjugated polysaccharide vaccines reveals distinct antigen specific memory B cell repertoires
Source: PLoS One. 2017 Sep 14;12(9):e0183738. doi: 10.1371/journal.pone.0183738 (PMC5598952; doi:10.1371/journal.pone.0183738)
Supplement: S2 Table — After isolation of individual antigen-specific B cells, heavy and light chain sequences were isolated and expression vectors were developed. 293T cells were co-transfected with heavy and light chains from a single B cell. Lysates from 293T cells were captured with luminex beads coated with the PS4, PS14, PS6B or PS23F antigens, and probed with PE-conjugated anti-human Ig antibodies. PE fluorescence was measured. Fluorescence intensities (measured in arbitrary units) for each cloned antibody and each antigen are shown. (PDF) [file pone.0183738.s005.pdf]

| Recombinant Mabs Generated from 1-week Samples of Animal C20882 (Boosting by Conjugated Vaccine) | | | | | | | | | | | | |
|---|---|---|---|---|---|---|---|---|---|---|---|---|
| Sample | PS-specific | isotype | PS4 | | PS6B | | PS14 | | PS23F | | Total Events | |
| C20882-B3-PS14+ | PS14 | IgG | 52 | 54 | 35 | 32 | 248 | 255 | 77 | 74 | 796 | 620 |
| C20882-D8-PS14+ | PS14 | IgM | 53 | 55 | 36.5 | 32.5 | 323 | 287 | 85 | 74 | 163 | 533 |
| C20882-B2-PS14+ | PS14 | IgG | 54.5 | 52 | 37 | 35 | 47 | 46 | 79 | 75 | 661 | 218 |
| C20882-D6-PS6B+ | PS6B | IgG | 59 | 59 | 8852.5 | 20834 | 46 | 42 | 84 | 79.5 | 519 | 363 |

Supplemental Table 2
