# Supplementary material for: Longitudinal multiparameter single-cell analysis of macaques immunized with pneumococcal protein-conjugated or unconjugated polysaccharide vaccines reveals distinct antigen specific memory B cell repertoires
Source: PLoS One. 2017 Sep 14;12(9):e0183738. doi: 10.1371/journal.pone.0183738 (PMC5598952; doi:10.1371/journal.pone.0183738)
Supplement: S1 File — (PDF) [file pone.0183738.s008.pdf]

From: Cordes, Eileen
Sent: Tuesday, March 8, 2011 2:07 PM
To: Bielawne, Jennifer; Casper, Marty; Cooper, David; Cordes, Eileen; DiJoseph, John; Li, Julia; French, Roger; Froeschl, Kim; Krol, Katharine; Lucas, Judy; Lyman, Larry; Macur, Thomas; Maher, John; Miller, Kim; Mischler, Scott; Obregon, Jennifer; Orlando, Holly; Pellecchia, Dena; Phillips, Jackie; Rosfjord, Edward; Strohmeyer, Timothy; Yates, David W; Illenberger, Arthur; Illenberger, Deanne; Scully, Ingrid
Subject: Protocol Renewal Approval PR-300418-961.

3/8/2011

Protocol PR-300418-961, entitled Immunogenicity study of vaccine candidates in Cynomolgus macaques, has been approved and will expire 3/8/2012.
